# Journal of Foot and Ankle Research, one year on

**DOI:** 10.1186/1757-1146-2-31

**Published:** 2009-11-11

**Authors:** Mike J Potter, Hylton B Menz, Alan M Borthwick, Karl B Landorf

**Affiliations:** 1 School of the Health Sciences, University of Southampton, Southampton, UK; 2 Musculoskeletal Research Centre, Faculty of Health Sciences, La Trobe University, Bundoora, Victoria, Australia; 3 Department of Podiatry, Faculty of Health Sciences, La Trobe University, Bundoora, Victoria, Australia

## Abstract

*Journal of Foot and Ankle Research *was launched one year ago, and a number of its key achievements are highlighted in this editorial. Although the journal is underpinned by professional bodies associated with the podiatry professions in the UK and Australasia, its content is aimed at the wider foot and ankle research community. Nevertheless, the journal's achievements over the past year reflect the development of research in the profession of podiatry. From this perspective, the journal may be viewed as contributing to the overall attainment of some of the profession's key goals and strategic aims over the last decade, across the UK and Australasia. The journal has also witnessed policy changes in the last year, and these are discussed - notably, the decision not to accept case reports for publication. We also report on a few of the key metrics, providing readers with a summary of the journal's performance over the last year.

## Introduction

It is now one year since *Journal of the Foot and Ankle Research (JFAR) *was launched, and the editors are able to report positively on its progress. In that time, the journal has received, as demonstrated by the statistics below, a considerable range of research papers illustrating a wide diversity of relevant topics. The papers accepted for publication demonstrate the scope and range of research being conducted within the foot and ankle arena. It is certainly true that, to date, the majority of papers have been authored by researchers from within the podiatry profession. As the journal is funded by the Australasian Podiatry Council and the UK Society of Chiropodists and Podiatrists, this is perhaps hardly surprising. Nevertheless, it is far from exclusively podiatric research that features in its pages, a fact that reflects the wider aims of the journal. Yet, to pause for a moment on the state of research within podiatry, it is probably relevant to reflect on the upward trajectory of research in the profession, in terms of its profile, range and rigour.

Podiatric research has been a significant factor in ensuring that this journal is able to pursue one of its aims in becoming a truly international outlet. Credit for this trend is, perhaps, more difficult to attribute, although educational changes in the profession have almost certainly influenced the increase in the practice and profile of research. It is probably fair to say that research has assumed a greater priority across the allied health professions in Australasia and the UK over the last 20 years, illustrated by the volume and breadth of its published research, and it may not be coincidental that both nations have witnessed a significant change in their professional educational status over that time, both at undergraduate and postgraduate levels. Education in Australasian and British podiatry has not always been at graduate level, and it is, perhaps, easy to forget that graduate status in UK and Australian podiatry was introduced in the 1980s, and only fully replacing a vocational, clinically orientated, professional award by the early 1990s. A similar picture has characterised developments in New Zealand [1,2].

In the UK, the development of a degree programme at the Polytechnic of Central London in the mid 1980s signalled the start of the progression towards a fully graduate profession, and merits comment as a major landmark in the overall process [3]. In Canada the situation is more complex, where two Provinces employ the US podiatric medicine degree, whilst the majority of other Provinces recognise UK, Australasian and South African graduate BSc programmes, and, in Ontario, the Michener Institute now requires graduate entry to its advanced diploma in podiatric medicine [4]. Indeed, the advent of this journal was greeted enthusiastically by the Canadian Federation of Podiatric Medicine [5]. In the USA, DPM degrees have been in place since the 1960s [6,7], and although international comparisons are notoriously difficult to make [8], it is nevertheless clear that uniform educational uplift in podiatry is now evident across the Anglophone world.

Let us take the UK as an exemplar. What is clear is that none of these changes happened by chance - they were part of a clear strategic intention [9]. The National Health Service Executive Chiropody Task Force report of 1994 identified nine research priorities for podiatry [10], leading, in 1995, to the NHS Research and Development Programme inviting the King's Fund to consider ways in which the podiatry profession might be "encouraged to do more research" [11]. One result of this was the establishment of the national Podiatric Research Forum, and, by 2003, a research strategy for the Society of Chiropodists and Podiatrists, in which the acquisition of a professional journal with medical database listing was central [9]. A number of editorials in the UK podiatry journals continued to emphasise the importance of research to the profession [12-14], and the development of a medical database listed journal as a crucial component and indicator of progress [15,16]. There is little doubt that the advent of Masters degree programmes in podiatry also enhanced research output, and graduate status has led, inevitably, to further research doctoral degree studies, and opportunities for podiatrists to become full-time, funded researchers. *JFAR *is potentially one of the key outlets for the publication of podiatric research, and is one of only seven foot and ankle journals listed in the PubMed database.

## Why no case reports?

In our first editorial, we stated that *JFAR *would only publish case reports if they "provide unique or important additional insights into the causes or treatment of foot and ankle disorders" [17]. However, we have since changed this policy, and no case reports will be accepted for publication in the journal. Our reason for this is the success of the Cases Network [18], an international, open access platform which publishes two journals - *Cases Journal *[19] and *Journal of Medical Case Reports *[20] - both of which, as their titles suggest, exclusively publish case reports. *Cases Journal*, edited by the former editor of the *British Medical Journal*, Dr Richard Smith, will publish "any case that is ethical and understandable", and the eventual goal of the Cases Network is to develop a large, searchable database of thousands of cases from all fields of healthcare practice.

To support this worthwhile initiative, we urge our readers to submit their case report papers to *Cases Journal*. In order to facilitate *JFAR *readers' access to relevant case reports, we have established a *JFAR *blog [21], and all relevant papers published in *Cases Journal *or *Journal of Medical Case Reports *are now linked to the main *JFAR *webpage. At the time of writing this editorial, 40 foot and ankle case reports had been linked to the website, covering topics as diverse as foot and ankle trauma, congenital lower limb deformities and infectious diseases. Please note that because *Cases Journal *is published independently of *JFAR*, all submissions are subject to an article processing charge, which is currently £199/US$330/€230/AUD$350.

## Why publish study protocols?

Readers unaccustomed to study protocols may have been somewhat perplexed by two papers published in the journal that described the rationale and methods for two randomised controlled trials in detail, but provided no results [22,23]. BioMed Central journals have published several such papers, the justification for which has been described previously [24]. Briefly, study protocol papers serve three main purposes. Firstly, they help researchers (and other interested readers) keep abreast of major studies that are currently underway. This is important, as it may help prevent any duplication of research effort. Secondly, the peer review process of protocol papers can help improve study design prior to commencement of the trial. Finally, study protocols can be viewed as an extension of trial registration, which is now mandatory for clinical trials [25]. The basis of trial registration is to allow for comparison of what was originally planned by the researchers and what was actually done. This helps identify whether the target sample size was obtained, whether any *post-hoc *changes were made to the study design, and whether any unplanned statistical analysis (sometimes referred to as "data-dredging") was undertaken. The overall goal of publishing study protocols is therefore to improve transparency in the conduct of research and to minimise bias. In keeping with the recommendations of the International Committee of Medical Journal Editors, all clinical trials submitted to *JFAR *must be registered.

## Journal metrics

### Characteristics of submitted manuscripts

Between the launch of the journal on the 28^th ^of July, 2008 and when this editorial was written (28^th ^of July, 2009), *JFAR *had received 71 manuscripts. Of these, 36 were accepted for publication, 20 were rejected, 3 were withdrawn, and 11 are currently undergoing peer review. The acceptance rate during the first year of the journal was therefore 51%. Of the published manuscripts, there were 25 original research papers, 5 reviews, two study protocols, two commentaries, one methodology article and one editorial. In September 2008, we also published a supplement containing abstracts of papers presented at the 1^st ^Congress of the International Foot and Ankle Biomechanics Community [26].

Published manuscripts represented the full spectrum of topic areas we originally envisaged in our first editorial [17], namely diabetology, paediatrics, sports medicine, gerontology and geriatrics, foot surgery, dermatology, wound management, rheumatology, diagnostic imaging, biomechanics and bioengineering, orthotics and prosthetics, and the broader areas of epidemiology, policy, organisation and delivery of services related to foot and ankle care. Although the majority of papers were from authors in Australia (15, or 43%) or the UK (13, 37%), reflecting the journal's society affiliations, we also published papers from authors in the USA (three) New Zealand (two), Denmark (one) and Spain (one).

### Most accessed papers

The *JFAR *website automatically tracks the number of accesses to each paper. For our first year of publication, the top ten most frequently accessed papers [27-36] are listed in Table 1. Each of these papers was accessed over 2,000 times, and it is worth noting that this only represents a fraction of the total number of accesses, as *JFAR *papers are also accessible as full-text through PubMed Central [37].

**Table 1:** Top ten most accessed papers, 28.7.2008 to 28.7.2009.

***Accesses***	***Paper***
4,126	Plantar calcaneal spurs in older people: longitudinal traction or vertical compression? (2008;1:7)
3,610	Arch height change during sit-to-stand: an alternative for the navicular drop test (2008;1:3)
3,540	Normative values for the Foot Posture Index (2008;1:6)
3,406	Effect of foot orthoses on lower extremity kinetics during running: a systematic literature review (2009;1:13)
3,070	Acral lentiginous melanoma of the foot and ankle: a case series and review of the literature (2008;1:11)
2,870	Musculoskeletal ultrasound imaging of the plantar forefoot in patients with rheumatoid arthritis: inter-observer agreement between a podiatrist and a radiologist (2008;1:5)
2,701	Growing pains: contemporary knowledge and recommended practice (2008;1:4)
2,221	Prevalence and correlates of foot pain in a population-based study: the North West Adelaide Health Study (2008;1:2)
2,189	Understanding the nature and mechanism of foot pain (2009;2:1)
2,175	Ultrasound evaluation of the abductor hallucis muscle: Reliability study (2008;1:12)

### Manuscript handling

When a manuscript is submitted to *JFAR*, it is initially reviewed by the editors, and if considered worthy of consideration, then undergoes the following processes:

(i) the manuscript is assigned to one of the editors, who is responsible for managing the peer review process;

(ii) two or three peer reviewers are contacted and invited to review the manuscript;

(iii) once the reviewers have accepted the invitation, they are sent the manuscript as a PDF file and are asked to complete the review;

(iv) completed reviews are sent to the authors;

(v) if the paper is considered to be worthy of consideration, the authors are asked to resubmit a revised version of the manuscript;

(vi) depending on the initial recommendation of the peer reviewers and the adequacy of the authors' responses, the manuscript is either editorially accepted, or sent for a second review (repeating steps iii to iv);

(vii) once accepted, the manuscript is forwarded to the editorial production team;

(viii) the editorial production team liaises with the authors to correct any formatting issues;

(ix) the manuscript is published as a provisional PDF file;

(x) the editorial production team liaises with the authors regarding the final html proof version of the manuscript;

(xi) the final PDF version of the paper is published.

Although the timing of many of these processes is under our control (e.g. assignment of the responsible editor, invitation of peer reviewers and forwarding of reviews to authors), many are not (e.g. the time taken for peer reviewers to reply to the initial invitation, time taken by peer reviewers to complete the review, and time taken by authors to respond to peer reviewer's comments). Nevertheless, the *JFAR *editorial team strives for rapid manuscript handling and peer review, and our goal is to have the peer review process completed within three months. For our first year of publication, the median time taken from the initial submission of the paper to the final editorial decision was 97 days, which indicates that we are on target to meet this goal.

### Website traffic

**Figure 1:**
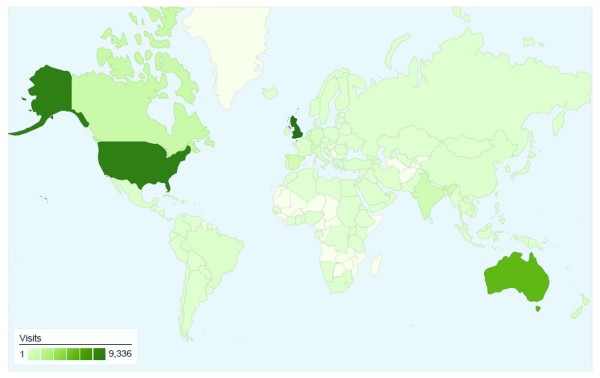
**Website accesses between 28.7.2008 to 28.7.2009 according to country (source: Google Analytics)**.

The magnitude and characteristics of traffic on the *JFAR *website have been tracked using Google Analytics [38] since November 2008. Over this time, there have been over 35,000 visits to the site from 151 different countries (see Figure 1). Most visits are from the UK (27%), followed by the USA (25%) and Australia (16%). The main source of traffic has been via Google searches (48%), followed by direct access (16%), the BioMed Central website (6%), PubMed (3%), Yahoo (3%) and Podiatry Arena (2%). On average, the site receives between 150 and 300 accesses per day.

## Thanks to our peer reviewers

All journals rely on the unpaid efforts of peer reviewers to assess the quality of submitted manuscripts. A list of peer reviewers who assisted the journal in its first year is provided in Table 2. We would like to thank them sincerely all for their hard work in ensuring the high quality of published manuscripts.

**Table 2:** Peer reviewers of manuscripts, 28.7.2008 to 28.7.2009.

***Reviewer***	***Institution***
Cedric Banfield	Cambridge NHS Trust, UK
Sue Barnett	University of the West of England, UK
Paul Bennett	Queensland University of Technology, Australia
Wanda Borges	New Mexico State University, USA
Catherine Bowen	University of Southampton, UK
Ivan Bristow	University of Southampton, UK
Alan Bryant	University of Western Australia, Australia
Joshua Burns	University of Sydney, Australia
Jackie Campbell	University of Northampton, UK
David Deberker	Bristol Dermatology Centre, UK
Sharon Dixon	University of Exeter, UK
Harriet Farquhar	Charles Sturt University, Australia
Jill Ferrari	University of East London, UK
Nicoletta Frescos	La Trobe University, Australia
Adam Garrow	University of Salford, UK
Mark Gilheany	La Trobe University, Australia
Jill Halstead	University of Leeds, UK
Farina Hashmi	University of Brighton, UK
Katarina Hjelm	University of Lund, Sweden
Sara Jones	University of South Australia, Australia
Anne-Maree Keenan	University of Leeds, UK
Tim Kilmartin	Derbyshire Country NHS Trust, UK
Michael Kinchington	Private Practice, Australia
Alberto Leardini	Instituto Ortopedico Rizzoli, Italy
Chris MacLean	Paris Orthotics, Canada
Xavier Martin	University of Barcelona, Spain
Ian Mathieson	University of Wales, UK
Tom McPoil	Northern Arizona University, USA
Hylton Menz	La Trobe University, Australia
Colin Morton	Falkirk Royal Infirmary, UK
Shannon Munteanu	La Trobe University, Australia
Susan Nancarrow	Sheffield Hallam University, UK
Deborah Nawoczenski	Ithaca College, USA
Cesira Pasquarella	University of Parma, Italy
Miguel Pons	Hospital Sant Raphael, Spain
Julia Potter	University of Southampton, UK
Trevor Prior	Homerton University Hospital, UK
Smita Rao	University of Iowa, USA
Anita Raspovic	La Trobe University, Australia
Lloyd Reed	Queensland University of Technology, Australia
Keith Rome	Auckland University of Technology, New Zealand
Dale Shuit	Governers State University, USA
Simon Smith	La Trobe University, Australia
Kate Springett	University of Canterbury, UK
Stephen Urry	Queensland University of Technology, Australia
Yosef Uziel	Meir Hospital, Israel
Scott Wearing	University of Strathclyde, UK
Anita Williams	University of Salford, UK
Matthew Young	Edinburgh Royal Infirmary, UK

## Authors' contributions

All authors assisted with drafting the manuscript, and all authors read and approved the final manuscript.
